# Supplementary material for: Asymmetric magma plumbing system beneath Axial Seamount based on full waveform inversion of seismic data
Source: Nat Commun. 2024 Jun 4;15:4767. doi: 10.1038/s41467-024-49188-y (PMC11535059; doi:10.1038/s41467-024-49188-y)
Supplement: Supplementary file 1 — Supplementary Information [file 41467_2024_49188_MOESM1_ESM.pdf]

Supplementary materials for “Asymmetric magma plumbing system beneath Axial Seamount based on full waveform inversion of seismic data”

Jidong Yang[1,*], Hejun Zhu[2,3], Zeyu Zhao[4,*], Jianping Huang[1,*], David Lumley[2,3], Robert J. Stern[2], Robert A. Dunn[5], Adrien F. Arnulf[6,7], and Jianwei Ma[4]

[1]National Key Laboratory of Deep Oil and Gas, School of Geosciences, China University of Petroleum (East China), Qingdao, Shandong, China
[2]Department of Sustainable Earth Systems Sciences, The University of Texas at Dallas, Richardson, TX, USA
[3]Department of Physics, The University of Texas at Dallas, Richardson, TX, USA
[4]School of Earth and Space Sciences, Peking University, Beijing, China
[5]Department of Earth Sciences, University of Hawaii, Honolulu, HI, USA
[6]Institute for Geophysics, University of Texas at Austin, TX, USA
[7]Present address: Amazon Web Services, CA, USA

Contents of this file

1. Figs. S1 to S2: Initial velocity and attenuation models as well as multi-channel seismic data used in viscoacoustic full-waveform inversion.

2. Figs. S3 to S9: Data fitting results and inverted P-wave velocity and Q models in three frequency bands. In 3-5 Hz stage, 32 iterations are applied, with early 15 iterations for simultaneously updating V_p and Q_p and later 17 iterations for updating Q_p alone. In 3-7 Hz stage, 43 iterations are applied, with early 15 iterations for simultaneously updating V_p and Q_p and later 28 iterations for updating Q_p alone. In 3-9 Hz stage, 30 iterations are applied, with early 15 iterations for simultaneously updating V_p and Q_p and later 15 iterations for updating Q_p alone.

3. Figs. S10 to S12: Numerical experiments of synthetic data for resolution analysis and quantitatively

error analysis. V_p and Q_p models are built according to the FWI results. The data processing workflow and FWI algorithm are the same as that for field data. After updating in three frequency bands, viscoacoustic FWI successfully resolves the low-velocity and low-attenuation anomalies. At the depth of 2.5 km below the seafloor, 600 m$\times$200 m velocity anomaly and 1.1 km$\times$300 m can be accurately imaged. Error analysis in Fig. S12 shows that relative V_p error is $\sim$10% at the model bottom while relative Q_p error is up to 20% near magma reservoir boundary. Local crosscorrelation between FWI models and the true models indicates that the structures of V_p and Q_p at a depth of 2 km below the seafloor are more reliable than those at greater depths (cross-correlation coefficient $>$80%).

4. Fig. S13: Estimated porosity (ϕ) models above the bottom of layer 2A from FWI Q_p model based on a exponential model as $Q^{-1} = Q_0^{-1}\exp(\beta\phi)$ with $Q_0 = 250$ and $\beta = 25$, and a linear model as $Q^{-1} = Q_i^{-1} + Q_p^{-1}\phi$ with $Q_i^{-1} = 0.0025$ and $Q_p^{-1} = 0.29$.

5. Fig. S14: Reflection image of the J48 survey line derived from reverse-time migration by ref.[1].

6. Figs. S15 to S19: Estimated temperature and melt fraction based on FWI V_p and Q_p models with different inclusion geometries.

7. Fig. S20: Exceedance graph illustrating the relationship between melt volume and partial melt fractions.

8. Fig. S21: 3D view for the main magma reservoir beneath Axial Seamount plotted according to the tomography velocity model in ref.[2].

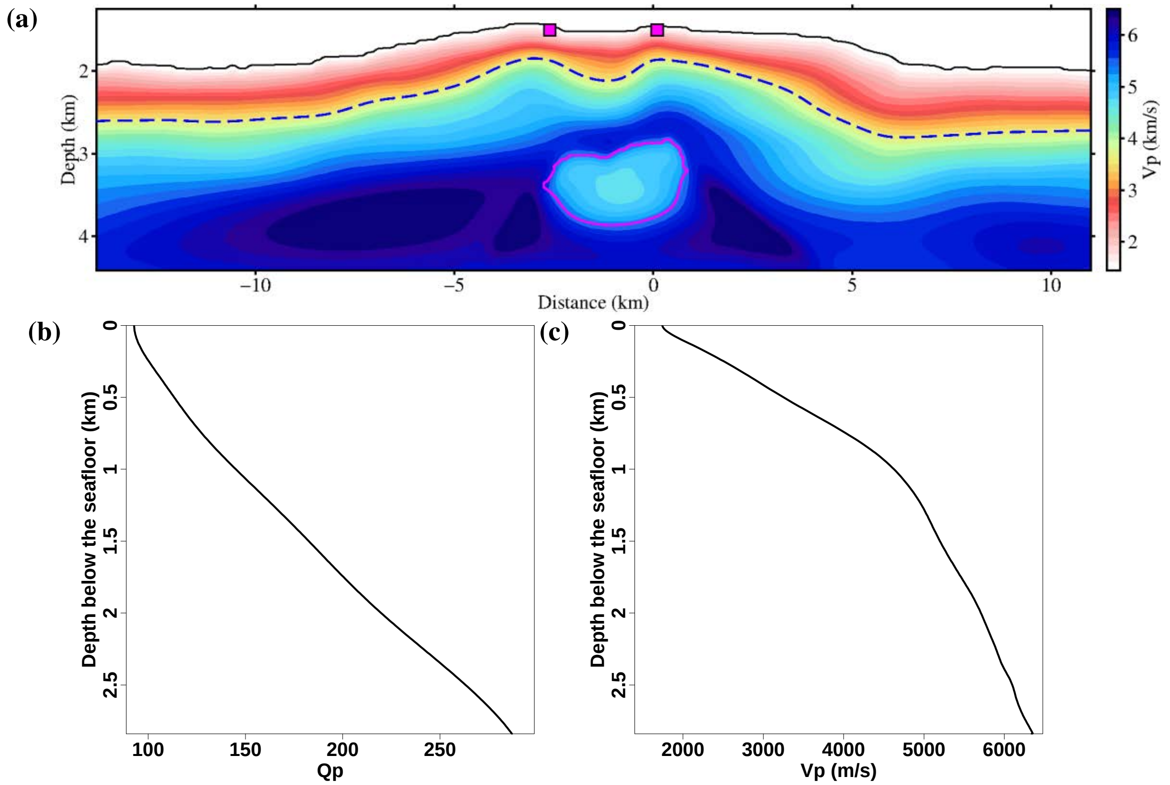

Fig. S1. Initial P-wave velocity (V_p) and quality factor (Q_p) models. (a) The V_p model along the J48 survey line is extracted from travel-time tomography[2]. The magenta polygon represents the main magma reservoir below the caldera, and the magenta rectangles on the seafloor denote the locations of hydrothermal vents close to the survey line. (b) A 1D quality factor (Q_p) model below the seafloor is built according to previous studies[3–5]. The quality factor defined as $Q_p = 2\pi \frac{E}{\Delta E}$ is used to characterize P-wave attenuation, where E denotes the total elastic energy in a wave cycle and ΔE denotes the dissipated energy in a cycle. The smaller the Q_p, the stronger the attenuation, and the larger the Q_p, the weaker the attenuation. In the ocean, we set Q_p as 1,000 assuming no attenuation for water. (c) 1D background V_p model used as a reference for computing velocity perturbations.

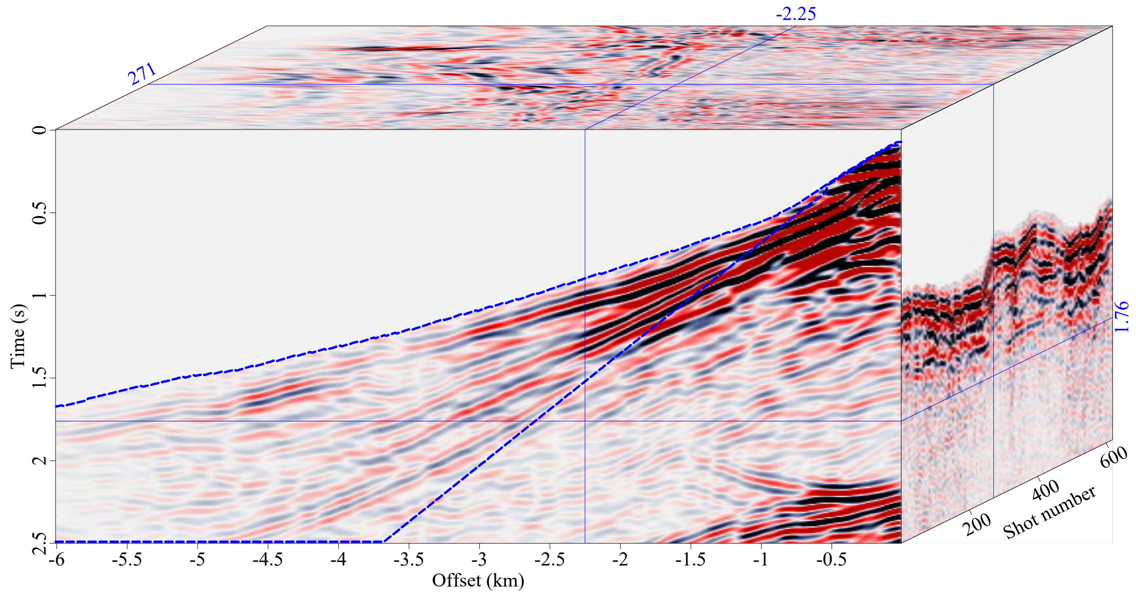

Fig. S2. Downward continued multi-channel seismic data used in this study. There are 619 common-source gathers, and each gather has 480 traces with a 12.5-m interval. The record duration is cut to 2.5 s from original 10.24 s. The blue dashed lines denote the time window used in FWI to avoid deep multiples and near-offset strong energy.

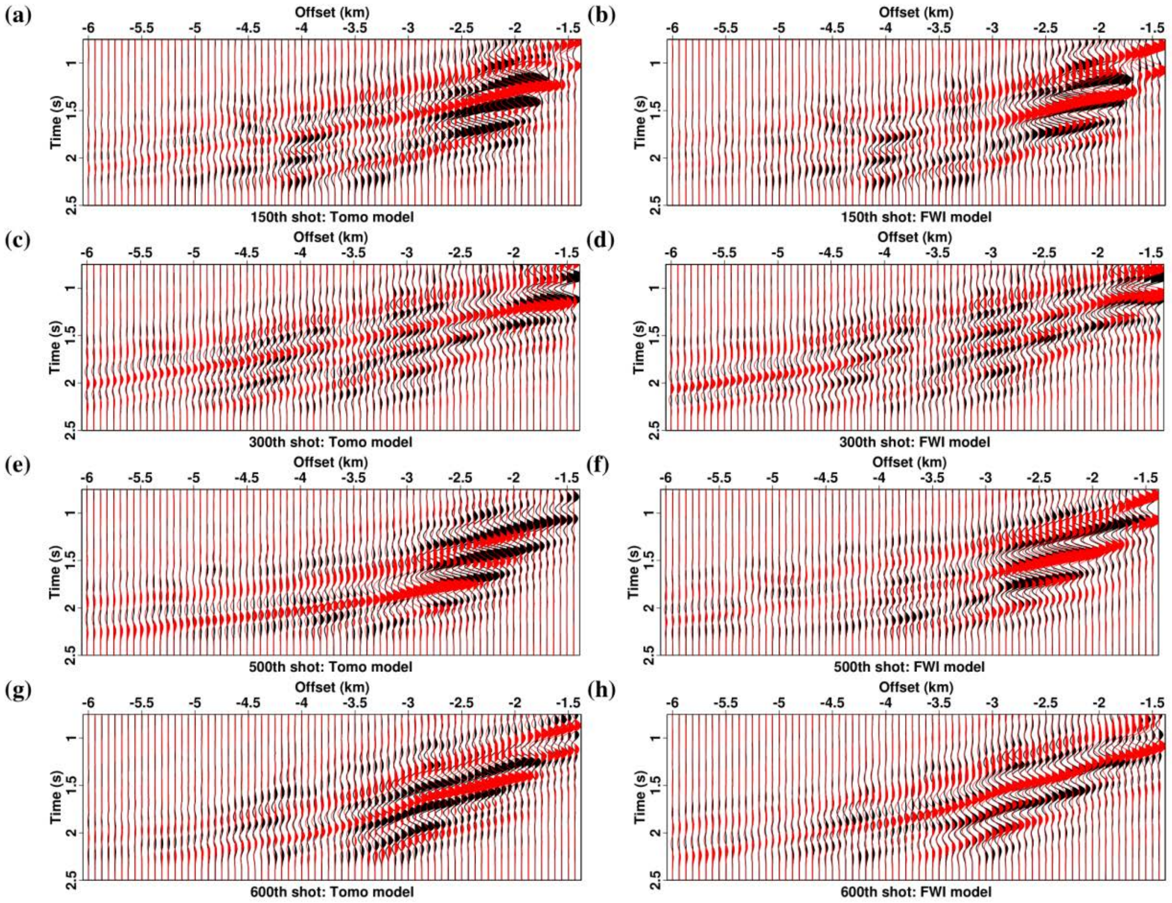

Fig. S3. 3-5 Hz data fitting between observed (black) and predicted data (red) for different shots. (a, c, e, g) Waveform comparisons for P-wave velocity model (Fig. S1) from traveltime tomography. (b, d, f, h) Waveform comparisons for the final velocity and attenuation models based on FWI.

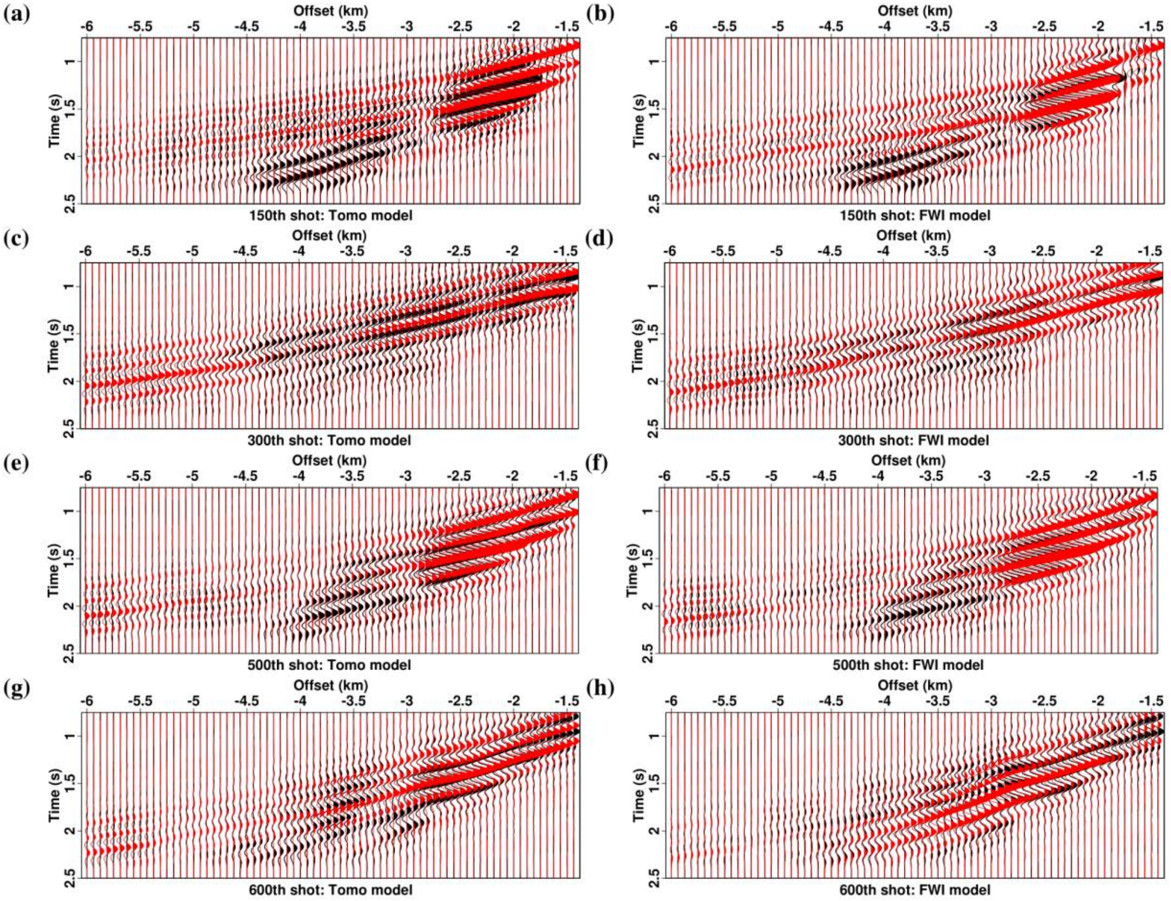

Fig. S4. 3-7 Hz data fitting between observed (black) and predicted data (red) for different shots. (a, c, e, g) Waveform comparisons for P-wave velocity model (Fig. S1) from traveltime tomography. (b, d, f, h) Waveform comparisons for the final velocity and attenuation models based on FWI.

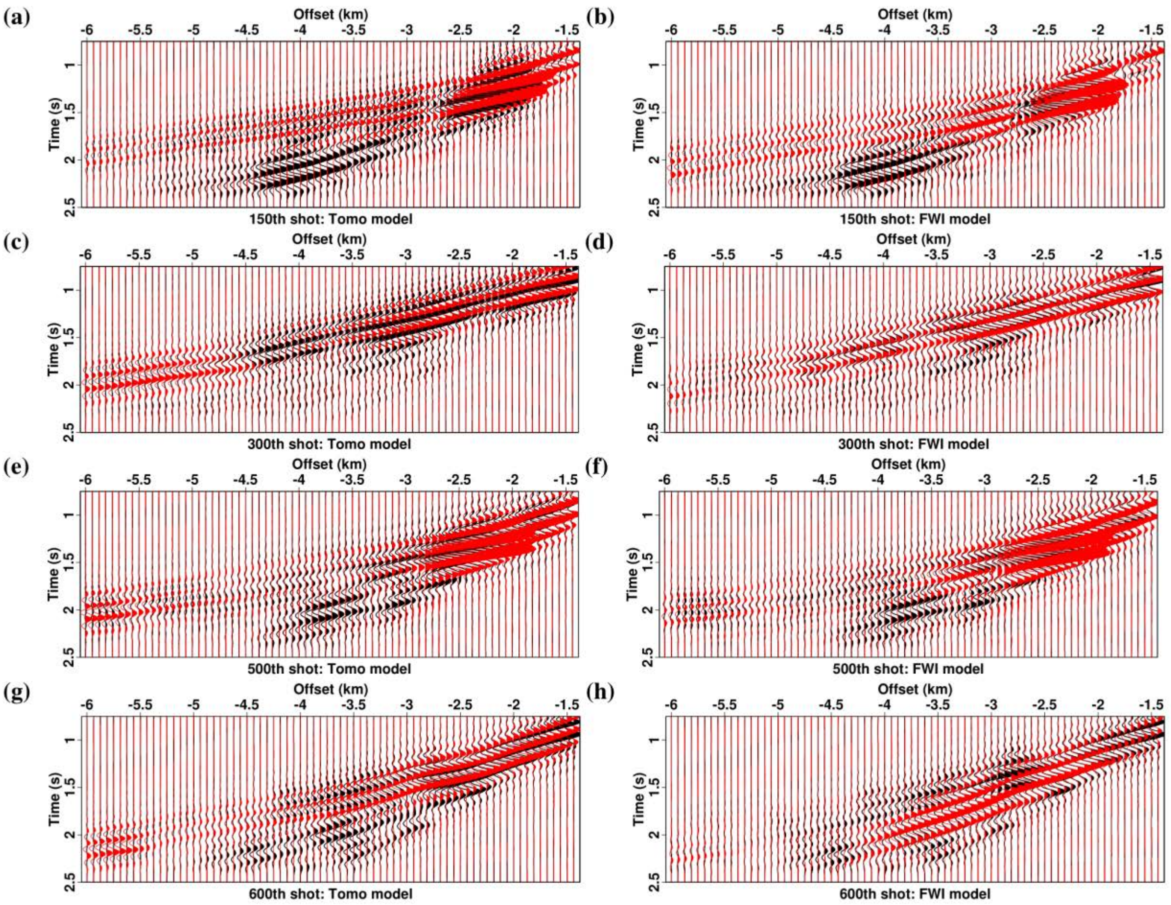

Fig. S5. 3-9 Hz data fitting between observed (black) and predicted data (red) for different shots. (a, c, e, g) Waveform comparisons for P-wave velocity model (Fig. S1) from traveltime tomography. (b, d, f, h) Waveform comparisons for the final velocity and attenuation models based on FWI.

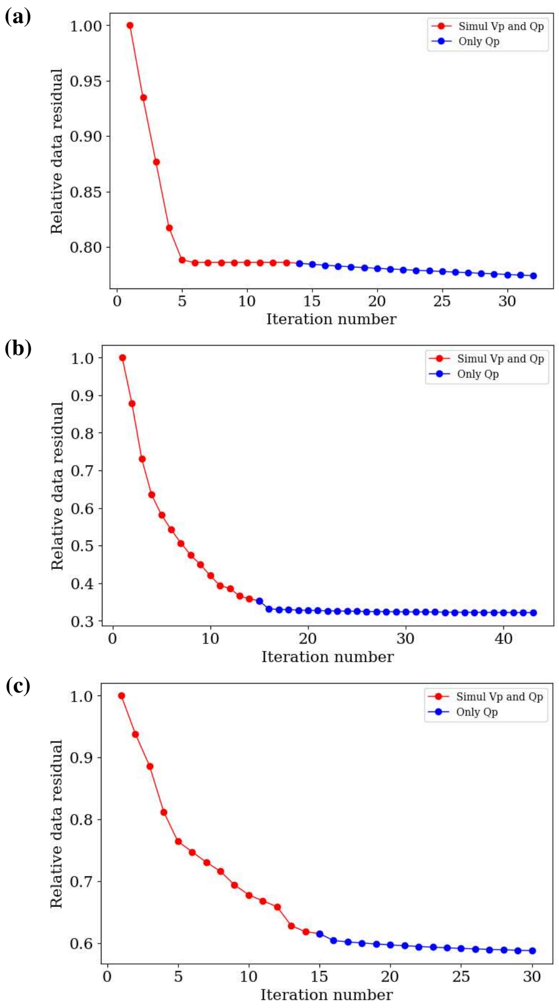

Fig. S6. Evolution of data misfits in three frequency bands. (a) 3-5 Hz, (b) 3-7Hz, and (c) 3-9Hz. Red dots denote the stage that simultaneously updates P-wave velocity and attenuation models. Blue dots denote the second stage that updates the attenuation model alone.

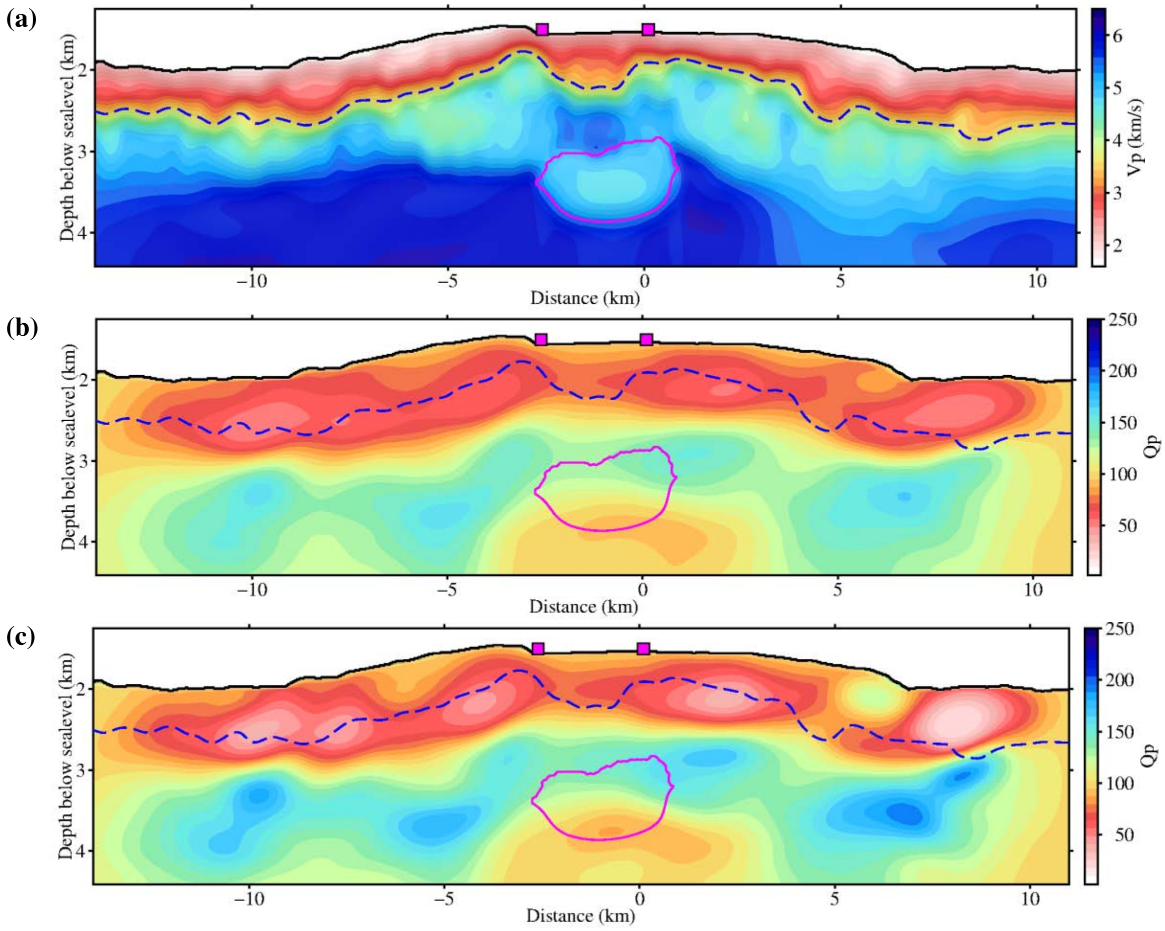

Fig. S7. P-wave velocity and attenuation models from 3-5Hz FWI. (a, b) Velocity and Q models at the first stage that updates two model parameters simultaneously (red dots in Fig. S6a). (c) Q model estimated after the second stage that updates Q model alone (blue dots in Fig. S6a).

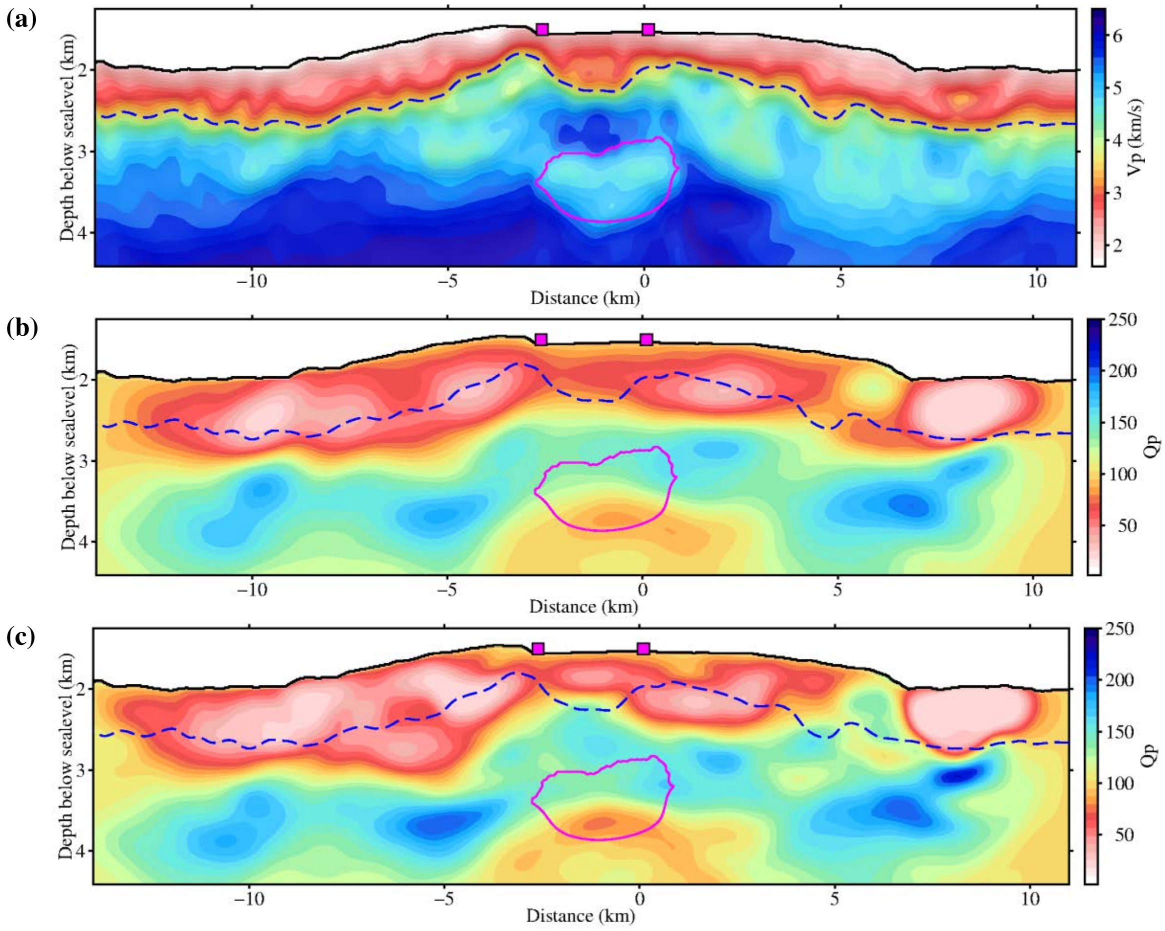

Fig. S8. P-wave velocity and attenuation models from 3-7Hz FWI. (a, b) Velocity and Q models at the first stage that updates two model parameters simultaneously (red dots in Fig. S6b). (c) Q model estimated after the second stage that updates Q model alone (blue dots in Fig. S6b).

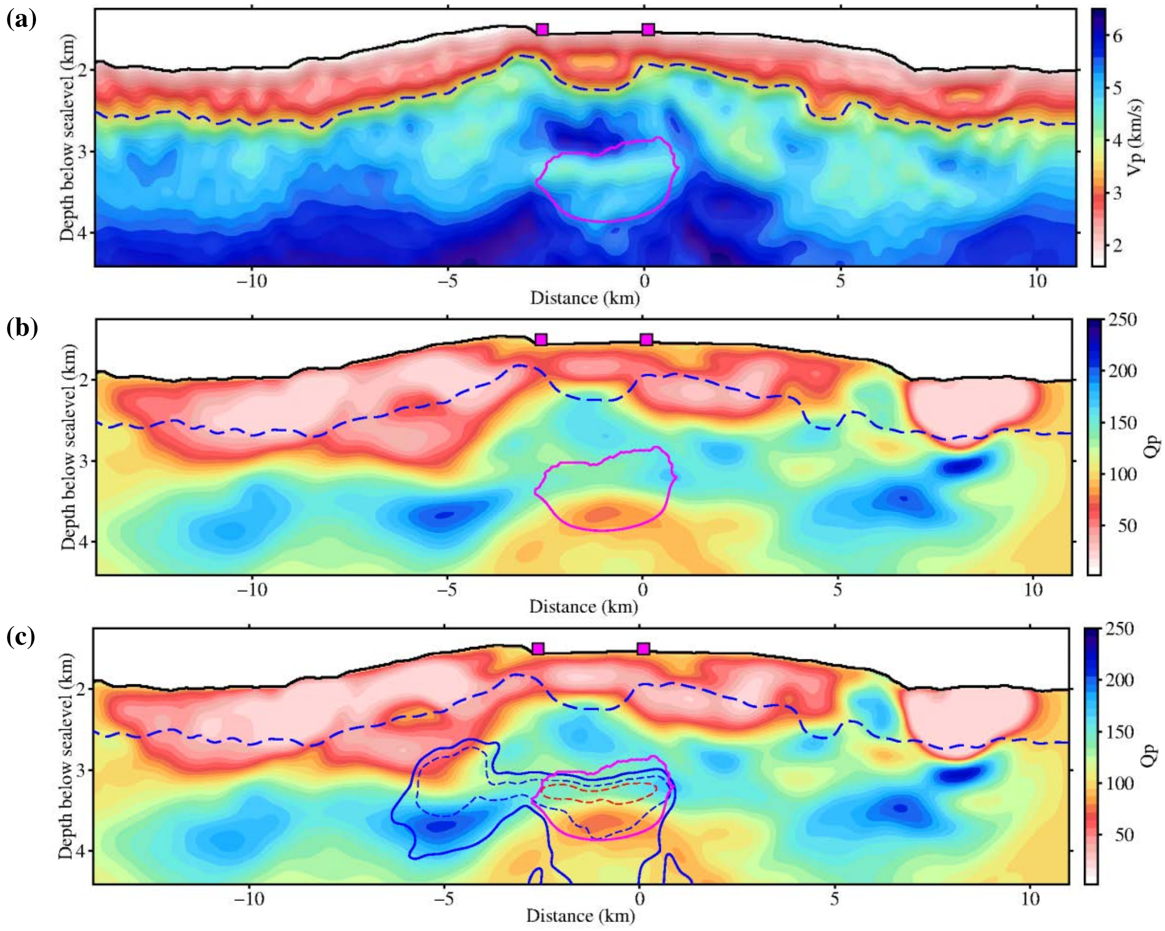

Fig. S9. P-wave velocity and attenuation models from 3-9Hz FWI. (a, b) Velocity and Q models at the first stage that updates two model parameters simultaneously (red dots in Fig. S6c). (c) Q model estimated after the second stage that updates Q model alone (blue dots in Fig. S6c). The solid blue contour in (c) outlines the western magma reservoir (WMR), main magma reservoir (MMR) and deep magma conduit. Dashed blue contour highlights the MMR and WMR, and red dashed contour denotes the upper MMR.

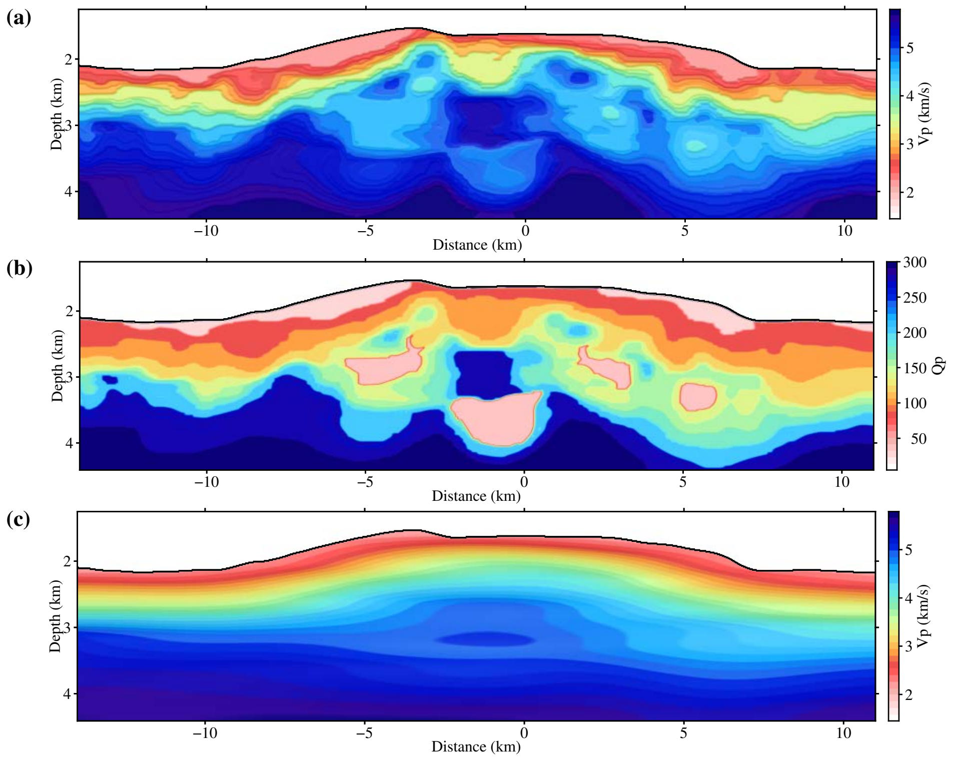

Fig. S10. P-wave velocity and attenuation models for synthetic tests. (a) The true P-wave velocity model, (b) the true Q model, and (c) the initial P-wave velocity model. The initial attenuation model is the same as shown in Fig. S1b.

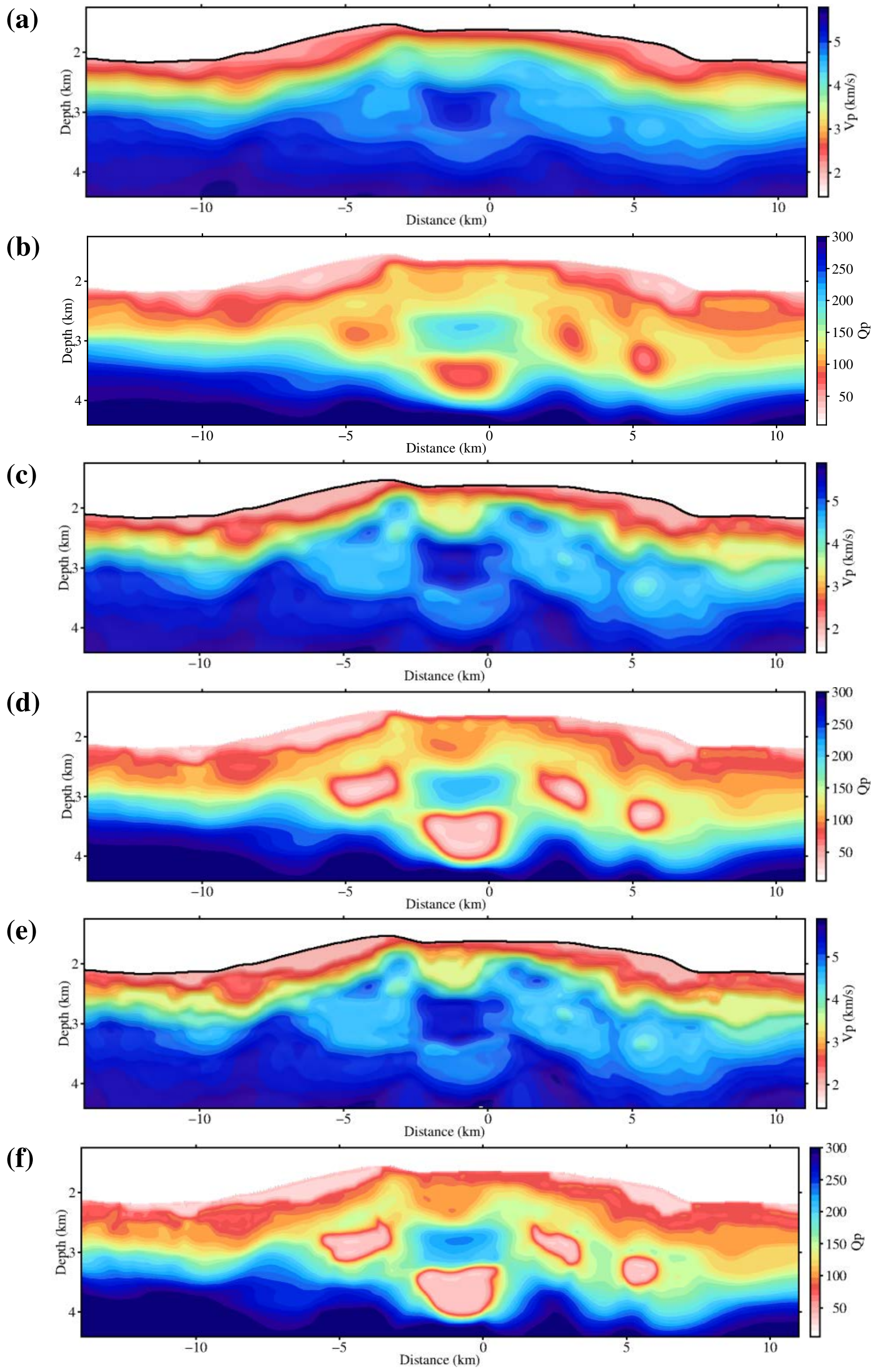

Fig. S11. FWI recovery results in three frequency bands for the synthetic tests. (a, b) P-wave velocity and Q models from 3-5 Hz data fitting, (c, d) P-wave velocity and Q models from 3-7 Hz data fitting, and (e, f) P-wave velocity and Q models from 3-9 Hz data fitting.

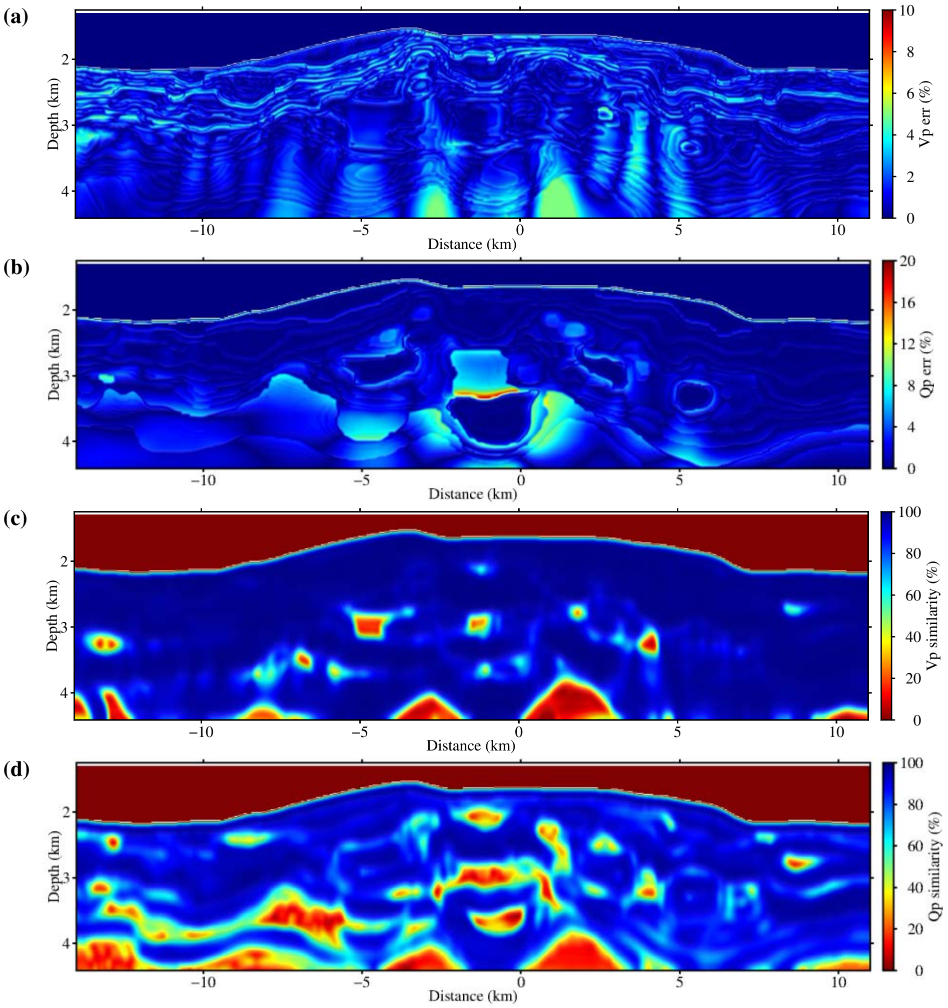

Fig. S12. Quantitative error analysis for the final FWI results in synthetic tests. (a, b) Relative errors of P-wave velocity and Q models, which are computed as $E_v = (v_p^{fwi} - v_p^{true})/v_p^{true}$ and $E_Q = (Q_p^{fwi} - Q_p^{true})/Q_p^{true}$, respectively. v_p^{fwi} and Q_p^{fwi} denote the FWI velocity and attenuation models. v_p^{true} and Q_p^{true} denote the true velocity and attenuation models. (c, d) Local crosscorrelation coefficients of P-wave velocity and Q models between FWI results (Figs. S11e, f) and the true models (Figs. S10a, b).

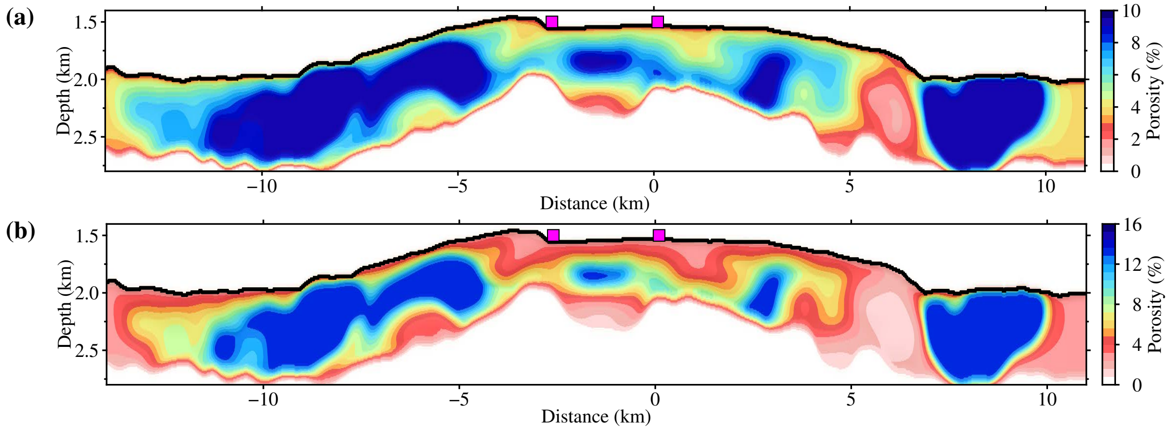

Fig. S13. Estimated porosity ϕ between the bottom of layer 2A and the seafloor according to the FWI Q model. (a) Porosity computed based on an exponential model[6], and (b) porosity estimated based on a linear model[6].

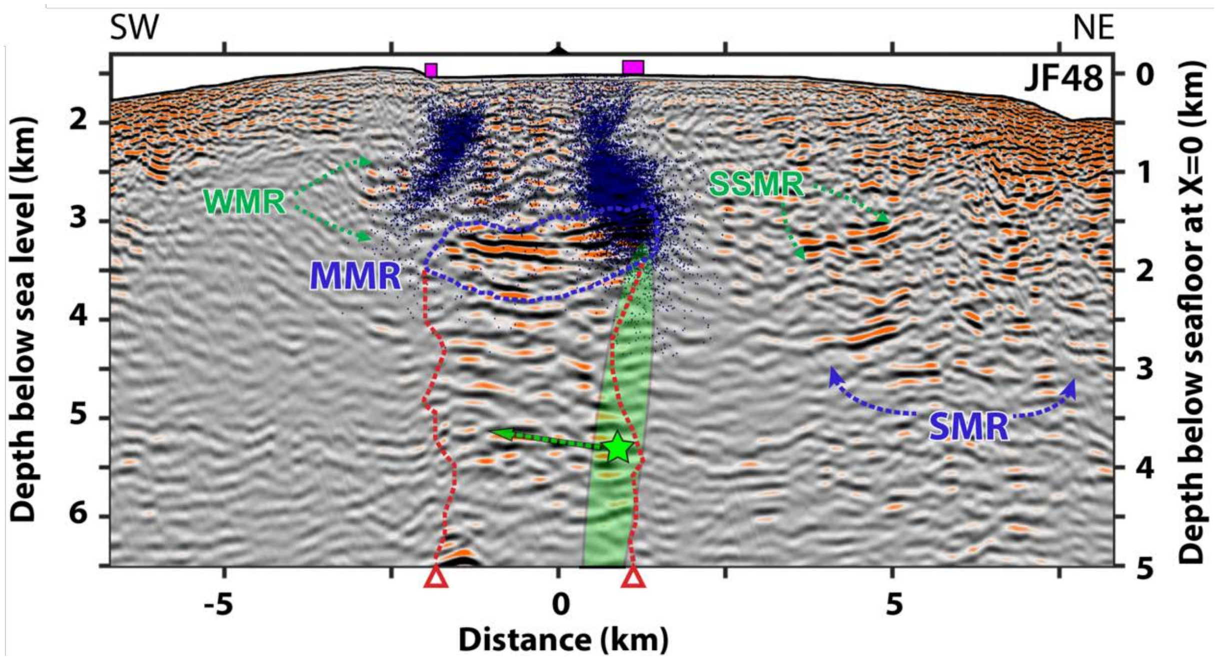

Fig. S14. Modified reflection image of the J48 survey line from reverse-time migration by ref.[1]. Strong reflection events are observed near the multiple magma reservoirs in the upper crust. The main magma reservoir (MMR) and secondary magma reservoir (SMR) are identified by ref.[2], and the western magma reservoir (WMR) and shallow secondary magma reservoir (SSMR) are identified in this study.

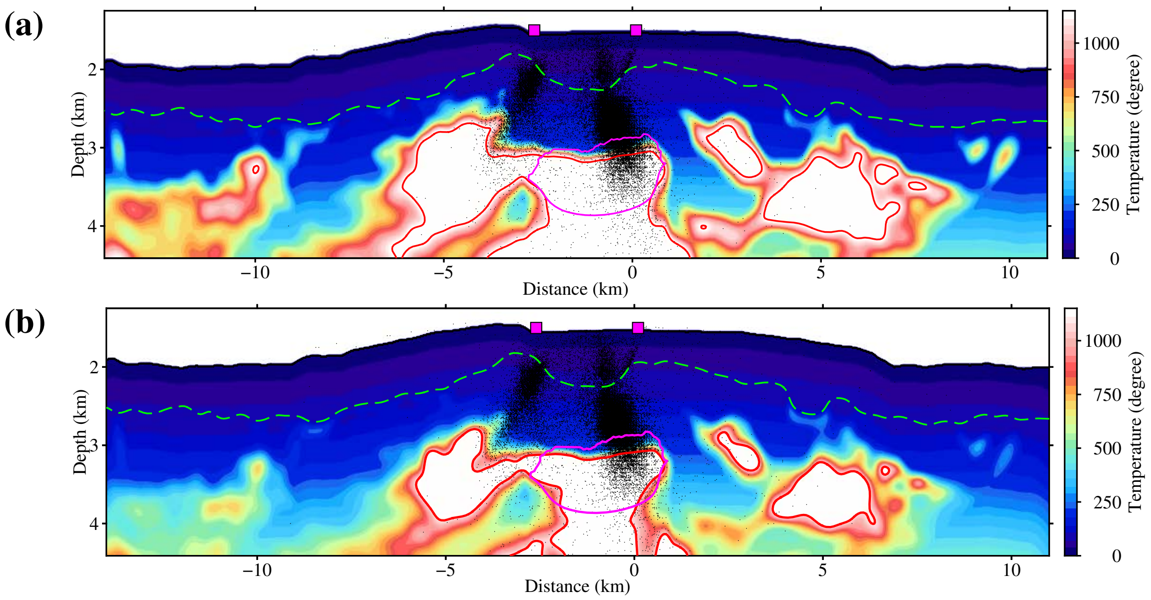

Fig. S15. Temperature estimated according to FWI V_p and Q_p models. (a) Temperature model computed by only accounting for anharmonic effect. (b) Temperature model computed by assuming both anharmonic and anelastic effects. The temperature above 1150°C is clipped. Blue dashed lines represent the the bottom of layer 2A. Black dots denote the hypocenters of earthquakes associated with 2015 eruption. Magenta polygon denotes the main magma reservoir (MMR) identified in ref.[7]. Magenta rectangles mark the locations of nearby hydrothermal vents.

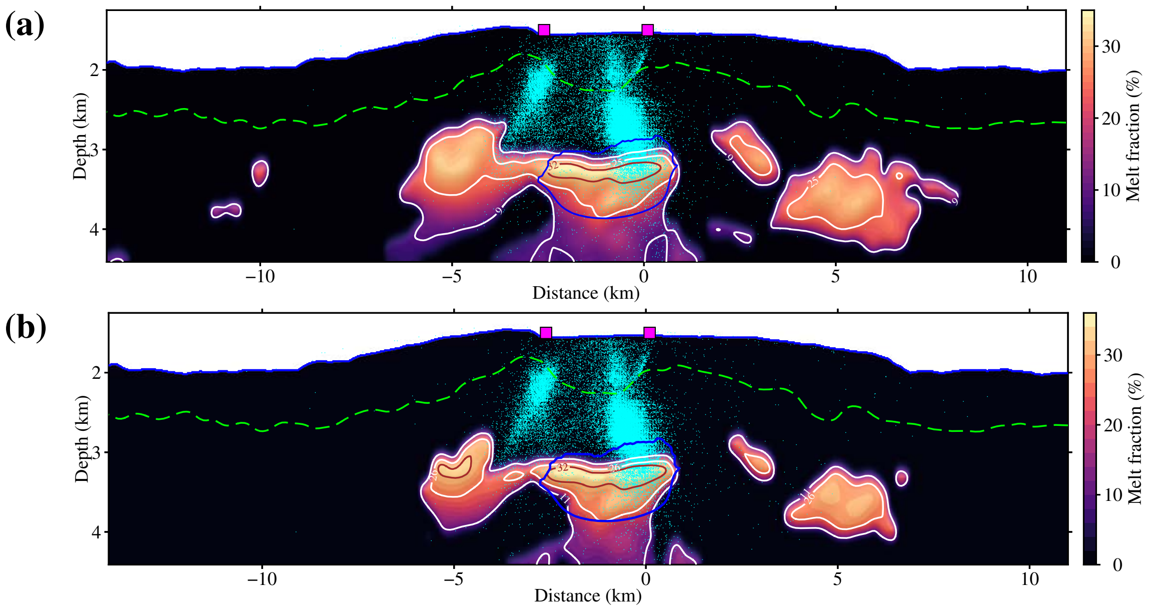

Fig. S16. Partial melt estimated assuming spherical inclusions based on a differential effective medium theory[2,8]. (a) Melt fraction model assuming the anharmonic effect alone. (b) Melt fraction model assuming both anharmonic and anelastic effects.

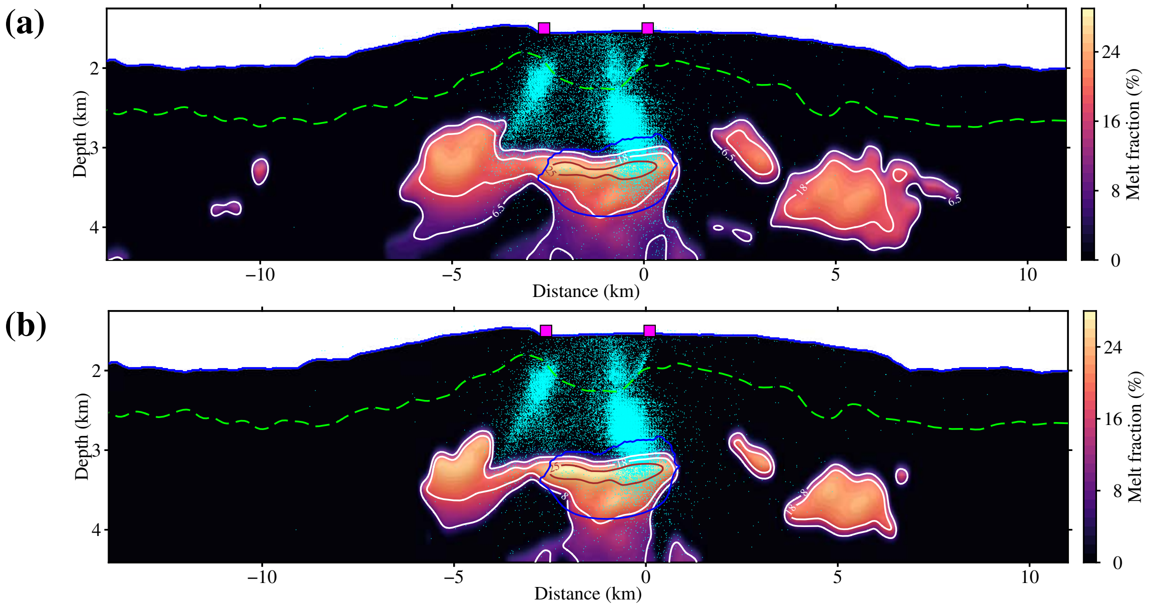

Fig. S17. Estimated partial melt assuming vertically elliptical inclusions with an aspect ratio of 0.5. (a) Melt fraction model assuming the anharmonic effect alone. (b) Melt fraction model assuming both anharmonic and anelastic effects.

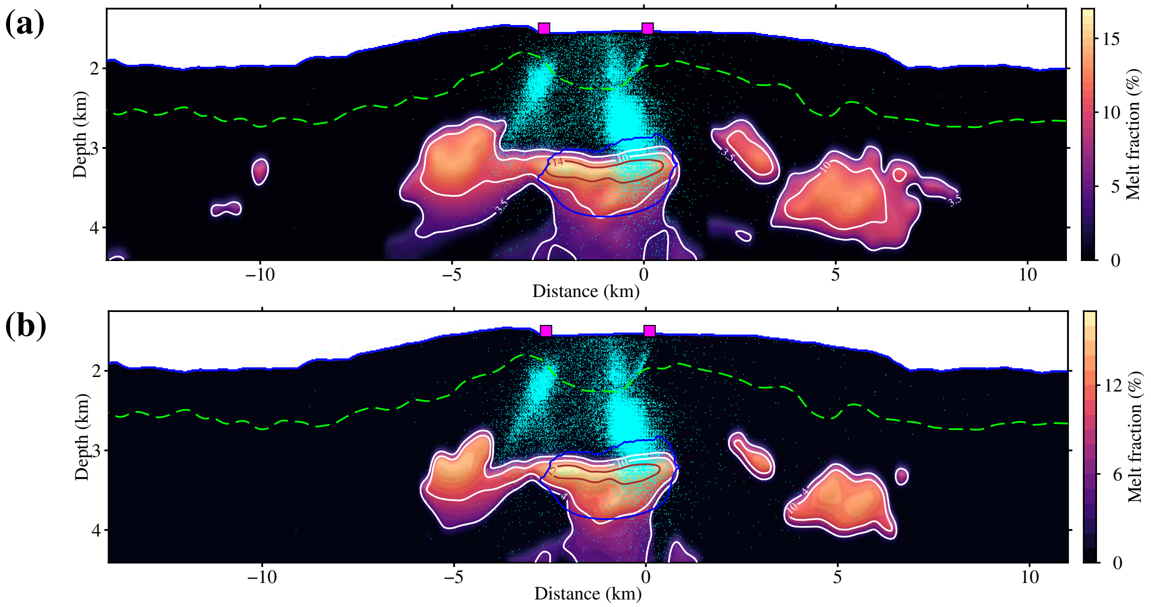

Fig. S18. Estimated partial melt assuming vertically elliptical inclusions with an aspect ratio of 0.1. (a) Melt fraction model assuming the anharmonic effect alone. (b) Melt fraction model assuming both anharmonic and anelastic effects.

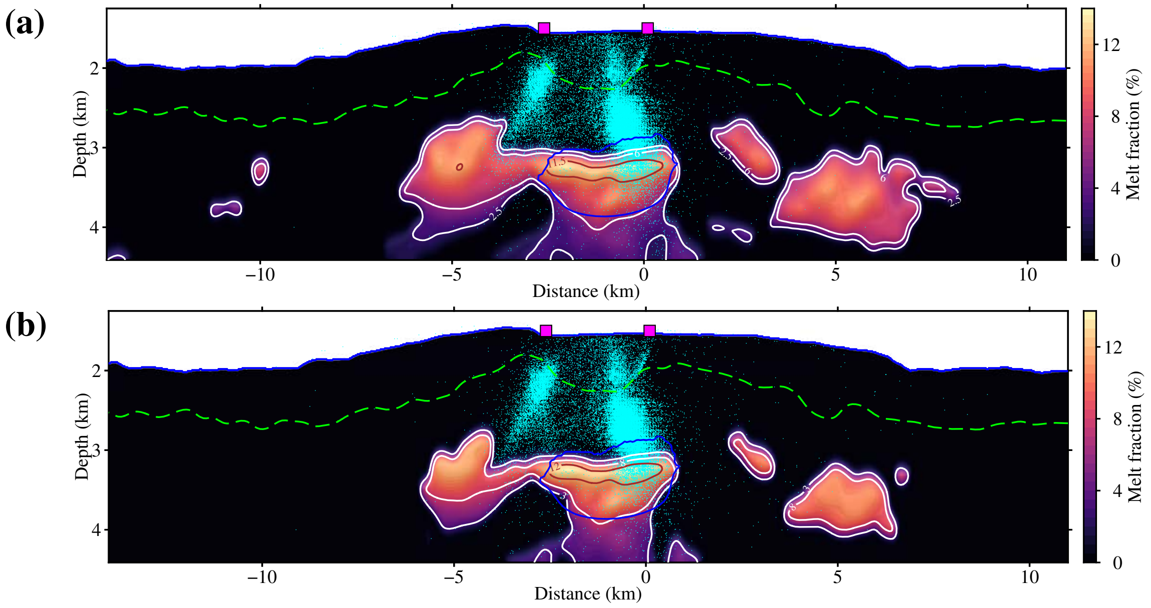

Fig. S19. Estimated partial melt assuming vertically elliptical inclusions with an aspect ratio of 0.02. (a) Melt fraction model assuming the anharmonic effect alone. (b) Melt fraction model assuming both anharmonic and anelastic effects.

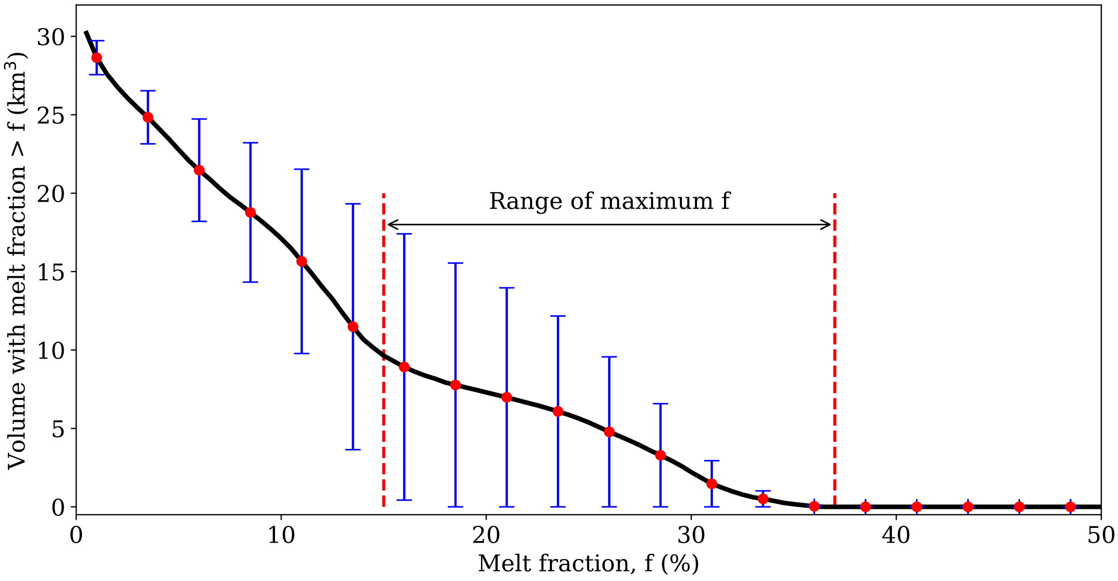

Fig. S20. Exceedance graph illustrating the relationship between melt volume and partial melt fractions. Error bars are based on two end-member partial models: one with spherical inclusion and the other with a vertically aligned elliptical body with an aspect ratio of 0.1. The fractions falling between the two dashed red lines represent the potential maximum partial melt range.

(a)

(b)

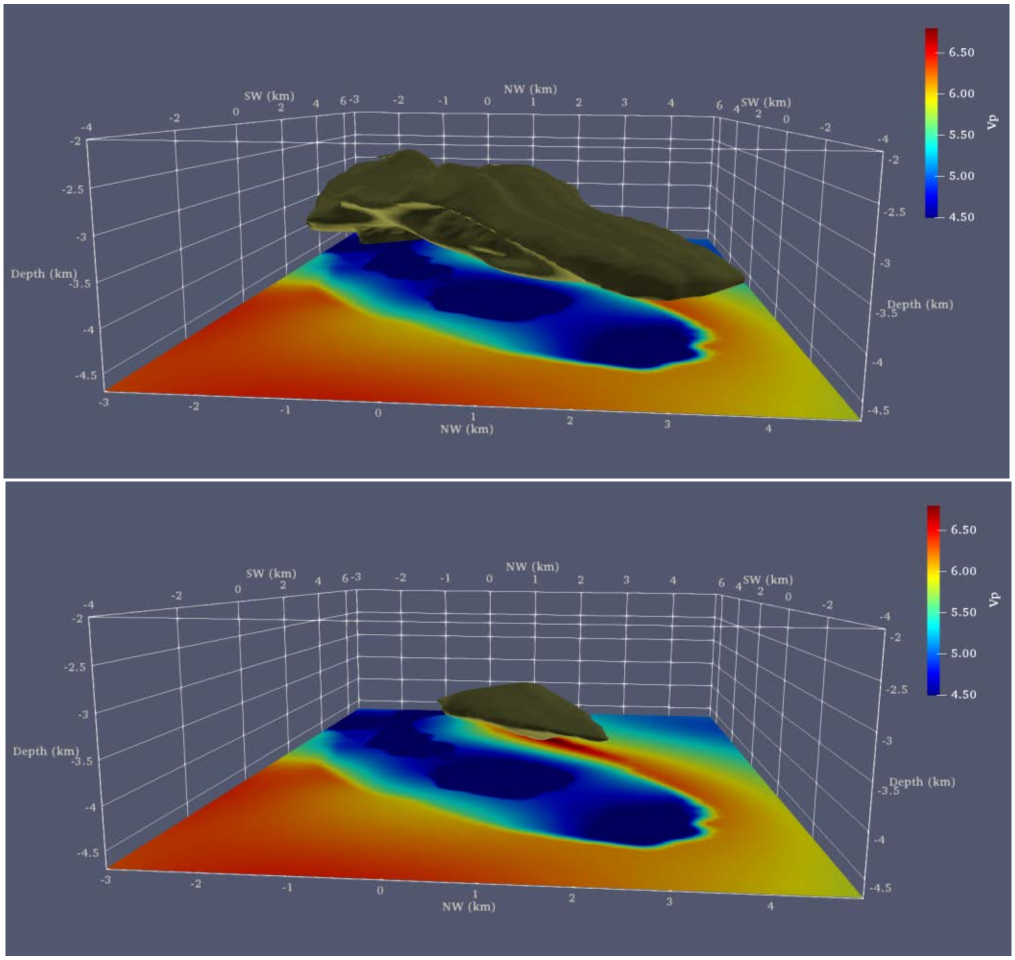

Fig. S21. 3D Geometry of the main magma reservoir (MMR) plotted based on the tomography velocity model in ref.[7]. (a) The isosurface of the whole MMR, and (b) the isosurface of the high-melt upper MMR. The slice at the bottom shows the P-wave velocity distribution at 3.5 km below the sea level.

References

1. Carbotte, S. M. *et al.* Stacked sills forming a deep melt-mush feeder conduit beneath Axial Seamount. *Geology* **48**, 693–697 (2020).

2. Arnulf, A. F., Harding, A. J., Kent, G. M. & Wilcock, W. S. D. Structure, seismicity, and accretionary processes at the hot spot-influenced Axial Seamount on the Juan de Fuca Ridge. *J. Geophys. Res. Solid Earth* **123**, 4618–4646 (2018).

3. Wilcock, W. S. D., Solomon, S. C., Purdy, G. M. & Toomey, D. R. The seismic attenuation structure of a fast-spreading mid-ocean ridge. *Science* **258**, 1470–1474 (1992).

4. White, D. J. & Clowes, R. M. Seismic attenuation structure beneath the Juan de Fuca Ridge from tomographic inversion of amplitudes. *J. Geophys. Res. Solid Earth* **99**, 3043–3056 (1994).

5. Wilcock, W. S., Solomon, S. C., Purdy, G. & Toomey, D. R. Seismic attenuation structure of the East Pacific Rise near $9^\circ\ 30'$ N. *J. Geophys. Res. Solid Earth* **100**, 24147–24165 (1995).

6. Goldberg, D. & Sun, Y.-F. Seismic structure of the upper oceanic crust revealed by in situ Q logs. *Geophys. Res. Lett.* **24**, 333–336 (1997).

7. Arnulf, A. *et al.* Anatomy of an active submarine volcano. *Geology* **42**, 655–658 (2014).

8. Berryman, J. G., Pride, S. R. & Wang, H. F. A differential scheme for elastic properties of rocks with dry or saturated cracks. *Geophys. J. Int.* **151**, 597–611 (2002).
